# Adoption of Precision Farming Tools: The Case of Italian Farmers

**DOI:** 10.3390/ijerph17030869

**Published:** 2020-01-30

**Authors:** Yari Vecchio, Giulio Paolo Agnusdei, Pier Paolo Miglietta, Fabian Capitanio

**Affiliations:** 1 Department of Veterinary Medical Sciences, University of Bologna, 40064 Ozzano dell’Emilia (Bo), Italy; yari.vecchio@unibo.it; 2 Department of Innovation Engineering, University of Salento, 73100 Lecce, Italy; 3 Faculty of Science and Technology, Free University of Bolzano/Bozen, 39100 Bolzano/Bozen, Italy; 4 Department of Agricultural Economics and Policy, University of Naples Federico II, 80055 Portici NA, Italy; fabian.capitanio@unina.it

**Keywords:** innovation process, awareness, complexity, precision agriculture, farming 4.0, Italy

## Abstract

The process of adopting innovation, especially with regard to precision farming (PF), is inherently complex and social, and influenced by producers, change agents, social norms and organizational pressure. An empirical analysis was conducted among Italian farmers to measure the drivers and clarify “bottlenecks” in the adoption of agricultural innovation. The purpose of this study was to analyze the socio-structural and complexity factors that affect the probability to adopt innovations and the determinants that drive an individual’s decisions. Preliminary results found high levels of adoption among younger farmers, those that had a high level of education, those with high intensity of information, with large farm sizes, and high labor intensity. A logit model was used to understand the role played by labor intensity and perceived in the adoption process. In light of the Common Agricultural Policy Reform post 2020, the findings suggest relevant policy implications, such as the need to increase awareness of PF tools and foster dissemination of information aimed at reducing the degree of perceived complexity.

## 1. Introduction

Precision Farming (PF) or “site specific management” emerged in late 1980s as a way to “do the right thing, at the right time, at the right point” [1]. PF employs a large set of innovation technologies such as sensors, information systems, enhanced machinery, and informed management [2]. The combined use of these tools allows for the monitoring of the temporal and spatial variability in the field, by adapting inputs to the real needs of the soil and the cultivated plants. Applications of site-specific management lead to an increase in profitability, improvements in the yield quantity and quality, a reduction in costs and environmental impacts [2,3,4]. 

PF tools, commercially available since the 1990s, provide a considerable support to farm management in various fields such as crop farming, horticulture, viticulture and zootechnics [5,6] and contribute to the Climate Smart Agriculture framework launched in 2009 which addresses the complex issue of how to achieve sustainable agricultural growth for food security under climate change [7,8]. Despite these applications, a low rate of adoption in Europe demonstrates that PFTs are applied less frequently than expected [9,10] due to strong barriers [11]. Adoption is not an immediate activity but depends on a large range of variables such as farmer characteristics, farm structure, location, organizational and institutional factors and those related to information. This complex scenario employs a widely explored conceptual framework of drivers and barriers. 

As reported by many studies, it is mostly young, well-educated and full-time farmers in large farms that are interested in PF. The highly recognized barriers are the high initial investment of PF equipment and high learning costs. Precision farming tools (PFTs) require high level of capabilities and skills to manage the large amount of information (big data) collected from PF tools [11,12,13,14,15]. In actual agricultural innovation systems, PF assumes the meaning of information-based management and characterizes a technological phase called Farming 4.0. 

Precision farming was the main objective of the European Commission legislative proposal published on 1 June 2018 in view of the post-2020 Common Agricultural Policy (CAP), having captured the attention of political decision-makers [16]. Following Rogers’ theory of “Diffusion of innovations”, this study fits into the furrow of scientific research concerning the analysis of the individualistic aspect of farmers in the adoption of PFTs [17,18,19,20,21].

In line with the above-mentioned studies, and to fill a gap in the literature, the present study aims at (i) analyzing the factors/drivers that stimulate an aware farmer, as defined by Adinolfi et al. [22] and Vecchio et al. [23], to adopt PFTs and (ii) determining the barriers that prevent the adoption process.

## 2. The Evolution of Innovation Process in the Agricultural Context

Klerkx et al. [18] identified four main approaches to agricultural innovation. Technology Transfer (TT), a technology-oriented approach, characterized the period of agricultural modernization (the 50s–80s). TT reflects the idea of knowledge transfer taking place through processes of a “top-down” type from researchers to farmers. In this period, the researchers’ main purpose was to enable rapid technological progress and increase agricultural productivity. This approach was strongly disconnected from the socio-political and institutional contexts where new technologies were operating [18,24]. 

After this period, the researchers’ studied in depth system-oriented approaches, such as Farming Systems (FS), Agricultural Knowledge and Information System (AKIS), and Agricultural Innovation System (AIS) [25,26,27].

The lack of attention to specific contexts (socio-economic, cultural and agro-ecologic) was observed in the 80s within the FS approach. This approach attributed a new role to farmers, which shifted from simple users to adopters of knowledge and technologies [18,24,28,29,30,31,32]. In the 90s, the transition from the top-down to bottom-up approach reflected in AKIS, where mechanisms of innovation were no longer considered a simple transfer of technology, but the exchange of knowledge and information between actors. The increasing importance attributed to the institutional and political components of the process of innovation also fostered a broader vision of the innovation system, in which the AKIS is a sub-set of a more complex framework, named AIS [18,33]. Compared to AKIS, AIS highlights the institutional and political dimensions of the innovation processes [18,34,35]. The goals of this system were to optimize the exchange of knowledge and interactions between actors and institutions that modeled the innovation process inside and outside the agricultural sector [36,37,38,39]. From this new conceptualization, different paths of analysis were developed. Vellema [40] describes it statically as “innovation support infrastructure”. A more dynamic analysis is provided by Hall and Clarks [41] and Klerkx et al. [37], where innovation systems are a co-evolution and networking process connected to the development of emerging technology (novelty) in a dominant production system. Hekkert et al. [42] proposed a third interpretation, no longer focused on the structure of innovation systems, but on the dynamics of innovation processes (labelled as “functions of innovation systems”) at the micro level. Starting from this view, a clear understanding of innovation mechanisms and the heterogeneous role of actors is required. From the description of the AIS approach, it emerged that new proposals such as learning platforms and networks could be the key to creating a scenario conducive to innovation, stimulating interactions and bringing further innovations to the agricultural sector [19,37,43,44,45,46,47,48,49,50,51,52].

## 3. Precision Farming Adoption Process 

In the context of agriculture, Rogers’ theory of innovation provides the most supported and influential theoretical basis for the technology adoption process [21]. Rogers’s view of the innovation process comprehends three stages: innovation invention, development, diffusion and adoption. In his theory, adoption is described as a sub-process of diffusion. The adoption process refers to the individual’s decision to integrate (or not) innovation into his or her life and “diffusion describes the adoption process across a population over time” [9,53]. Rogers [54] identified four components of the process. The first component is innovation itself, characterized by five attributes: relative advantage (perception of an individual that innovation will be better or worse than similar ideas), trialability (degree of experimentation with the innovation), observability (perception of how available and visible an innovation is to an individual), complexity (perception of how difficult to comprehend what an innovation is), and compatibility (perception that a particular innovation is similar to existing or past ideas). The second refers to communication channels, mechanisms by which information about innovation passes from one individual to others. The third element is the social system that refers to the context, culture and environment in which innovation operates and individuals are involved. The fourth component is time. The diffusion process, represented as a normal curve, identifies different types of adopters [9]: innovators, early adopters, early majority, late majority, late adopters or laggards [55,56]. Categories of adopters differ for socio-economic characteristics, personality variables and communicative behaviors. It is assumed that innovators are risk and change takers; the late majority are skeptical, and laggards are traditionalists. Among the socio-economic characteristics of innovators/early adopters, there is a higher level of education, high social status, and larger and more specialized activities [14,54,55]. Furthermore, they are more rational, oriented towards obtaining results, have a higher degree of social participation, and have more contacts with technical assistants and access to information [55]. When an individual approaches a new idea, he hardly adopts it immediately. The adoption decision is, in fact, preceded by a learning period in which an individual acquires information or eventually experiments with the innovation for a limited period of time [2,57,58]. The decision to adopt is not a discrete event, but the result of a multistage process [59,60]. Rogers [54] 1995) recognized five stages of the adoption process: (i) awareness: the individual learns about the existence of the new practice/idea; (ii) interest: the individual develops an interest and seeks more information on it; (iii) evaluation: the individual mentally applies it to his own context; (iv) trial: the individual applies it, usually on a smaller scale; (v) adoption: the individual decides on a continued use of the innovation in the future. Numerous criticisms were raised against this model with regard to serious shortcomings reported in the field of scientific validation [61]. First of all, there is no clear scientific evidence that the adoptive behavior (early adopter, later adopter, etc.) remains completely coherent over time. The characteristics of the innovation cannot explain the adoptive behavior of individuals because they influence the adoption process based on how they are perceived by individuals [62,63]. These criticisms led Rogers and Schoemaker [64] and later Rogers [54] to modify the description of the adoption process, suggesting the “decision-innovation process” as an alternative. This process is divided into five phases: (i) knowledge (the individual is first exposed to the innovation and receives some information on it); (ii) persuasion (the individual forms a favorable or unfavorable attitude towards innovation, by gaining enough knowledge about the innovation’s characteristics); (iii) decision (the individual decides to adopt or not, weighing advantages/disadvantages of using innovation), (iv) implementation (the individual employs the innovation to a varying degree depending on the situation), (v) confirmation (the individual reflects on his or her decision and re-evaluates whether to continue or not). 

The two processes, the first theorized in 1962 and the second in 1995, both underline as a first stage the phase in which the individual first “heard of” the existence of new technologies. It seems logical that this phase can be described as the “awareness phase”. The “knowledge phase” comes in a second stage, only after awareness, through a learning process in which the farmer acquires information about the technology [57,59,65,66]. Some researchers [14,55,65,66,67,68,69,70,71] focus on weighing the role of awareness in adoption. Diagne [67] and Diagne and Demont [65], going beyond the “static” analysis of adoption, and found that being aware is a precondition of adoption. Diagne and Demont [65] and later Simtowe et al. [68] provide empirical evidence that “when a technology is new and the target population is not universally exposed to it, the observed sample adoption rate is not a consistent estimator of the true population adoption rate”. Daberkow and McBride [14] verified that stages are differently affected by farm and operator characteristics. Awareness and adoption (conditioned by awareness) are positively affected by farm size, education, full-time farming, and familiarity with computers. Their results show the “unaware” farmers manage small farms, are often less educated, are mostly older, and have less access to credit. Lambrecht et al. [71] consider “adoption, conditioned by awareness and try out phases” and find that younger farmers are more likely to “try out” a new technology, whereas older and more experienced farmers are well-oriented to the continued use (“adoption”), if they have already “tried it out”. There is a large overlapping between factors, affecting both awareness and adoption: they influence differently in every stage of the adoption process. This result show that factors hindering adoption in the initial phase of the diffusion cycle (early adopters) may not represent an obstacle later [72,73,74].

A large number of recent scientific literature identify multi-stakeholder engagement and networking, within Innovation Platforms (IPs), as the key factor to making farmers aware of and stimulating them to adopt new knowledge (technological or other) in the farming innovation development process [27,52,75]. Starting from these considerations, this study provides an empirical analysis of Italian farmers, considering awareness as a precondition of the adoption of precision farming tool with the aim to reveal and analyze both factors affecting the probability to adopt and determinants driving an aware individual not to adopt. 

## 4. Data Collection and Methods

This research comprised of a pilot study, aimed at identifying the behavioral, normative, and control beliefs likely to determine farmers’ intentions to adopt (or not) precision farming tools, and a main study aimed at investigating the contributions of each factor in affecting the PFTs’ adoption process.

### 4.1. Pilot Study

The pilot study is aimed at identifying the most important elements linked to farmers’ intentions to adopt (or not) precision farming tools. This exploration was conducted in line with the scientific literature in this field [76] through face-to-face interviews with a random sample of 35 farmers. Filling of an open questionnaire was supported by a research assistant.

During the interviews, the knowledge and beliefs that each farmer had regarding precision farming were investigated. The respondents indicated their opinion regarding: (i) the advantages and disadvantages of precision farming tools that could affect their decision to adopt them; (ii) the contextual conditions that may encourage vs. discourage their decision; and (iii) the events or situations potentially able to facilitate vs. hinder the adoption of precision farming tools. From the pilot study, it was possible to identify the dimensions of the main study analysis, which focused on the relationship between the farm’s socio-structural dimensions, the perceived complexity, and the adoption of precision farming tools. 

### 4.2. Main Study

#### 4.2.1. Sample

The main study was conducted on a non-random sample of farms operating in different Italian regions. The questionnaire was administered to over 200 farmers. Of the potential participants, 174 completed the questionnaire in full. This number is in line with previous literature [76,77] and can be considered valid for empirical analysis. Extant research has indeed ascertained that farmers are generally unwilling to spend their time completing surveys [77] and sharing data and/or information on themselves and their activities [4]. The sampling was carried out at national fairs dedicated to precision farming. Following the intentional sampling approach [78], we included in the survey only those farmers who affirmed that they knew precision agriculture so that they had answers that were influenced by knowledge of the subject. The limits of this approach were subjectivity and generalization [79], but it allowed the implementation of an exploratory analysis [80].

#### 4.2.2. Questionnaire

The questionnaire was structured in two sections. In the first part, the socio-structural dimensions of the farms that were found to be relevant to the pilot study survey were investigated, i.e. farmer’s age, farm size, labor intensity, intensity of information and level of education. In the second part, five questions were asked on different aspects concerning precision farming to build a variable of perceived complexity:

◾ efficiency effects: introduction of PFTs leads to efficiency gains at the farm level;
◾ institutional effects: introduction of PFT requires farmers’ engagement in stakeholders’ and networking platforms;
◾ organizational effects: introduction of PFT requires organizational and structural adjustments that are difficult to implement;
◾ effects on agricultural practices: PFT requires radical change in agricultural practices;
◾ financial exposure effects: introduction of PFT requires investments to be recovered in the long term.

The measure of farmer perception was done on a Unipolar Likert scale [81]. A 6-point unipolar Likert scale was used, with a value of 0 indicating “I do not agree, it is not an element of complexity” and 5 pertaining to “I agree, it is an element of complexity”. The maximum value achievable from the combined questions was 25. The variable was calculated based on the following Equation (1):

(1) $$y=\frac{\sum_{i=1}^{n}x_{i}}{25},$$

y assumes values between “0” and “1”. The value of “1” refers to the perception of high complexity by respondents.

Cronbach’s α was used to test internal consistency. This index is a measure of reliability of the test, that is, how a set of items are related as a group. A high value of this index does not necessarily ensure that the scale is unidimensional, but the latter could be tested through exploratory factor analysis. The function of standardized Cronbach’s α is as follows (2):

(2) $$\alpha =\frac{N\cdot \bar{c}}{\bar{v}+\left(N+1\right)\cdot \bar{c}}$$

where N is the number of the items, c is the average inter-item covariance among the item, and v represents the average variance.

In this study, there was no strong correlation (greater than 0.6) among the five elements of complexity, and the mono-dimensionality of the scales was tested through exploratory factorial analysis, which extracted only one factor. Subsequently, Cronbach’s α showed good results of consistency (over 0.81), which added to the result of factorial analysis, allowing us to affirm that the perceived complexity variable has been built on a robust quantitative justification.

#### 4.2.3. Logit Regression

Most ex-post papers on the adoption of precision technologies generally use logit models to explain the adoption behavior of farmers [12,13]. Logit regression analysis is employed when faced with the binary adoption choice and a small sample. Notwithstanding binary logistic regression modelling can be extended to categorical outcomes, using multinomial logistic regression. The principles are very similar, but with the key difference being that one category of the response variable must be chosen as the reference category. The analysis of this study is based on associating the different probabilities with which the modes of the dependent variable are presented, to the changing of independent ones. The logit model can be represented by three equations: predictive, stochastic, and systematic. The predictive part remains unchanged in the model, unlike the other components. The parameter to be estimated is $\eta_{i}$, where i corresponds to the N cases considered. It is calculated through a linear expression of K variables X, called regressors, as illustrated in Equation (3):

(3) $$\eta_{i}=\beta_{0}+\sum_{j=1}^{k}x_{ij}\beta_{j}$$

$\beta_{0}$ is the value of $\eta_{i}$ when all the regressors are equal to 0, whereas $\beta_{j}$ measures the variation of $\eta_{i}$ for each unit increasing with the corresponding regressor $x_{j}$. The stochastic component, on the other hand, varies in the model. The dependent variable y categorically imposes different assumptions on the random variable Y. In the case of binomial logistic regression, the variable dependent $y_{i}$ is binary. It is associated with a random variable $Y_{i}$, which has a Bernoullian distribution and is characterized by the parameter $\pi_{i}$, $\pi_{i}$ which indicates the probability that a certain event will occur; $\left(1-\pi_{i}\right)$ represents the opposite probability (4):

*y_i_* ∈ *Y_i_* ~ *Bernoulli* (*π_i_*) (4)

The systematic component, therefore, underlines the logistic function, which binds the probability distribution of $Y_{i}$ to independent variables and allows for the linking of the parameter to estimate $\pi_{i}$ to the predictive Equation (5):

(5) $$\pi_{i}=exp\left(\eta_{i}\right)/\left(1+exp\left(\eta_{i}\right)\right)$$

The β coefficient, which produces a variation of $\pi_{i}$ between 0 and 1, represents the parameter to be estimated and describes effects of the independent variables on the dependent one. The Wald test examines whether an independent variable has a statistically significant relationship, and therefore if there is an effect with the dependent variable. The Wald statistic is equal to the ratio between the logistic coefficient and its standard error, squared. To express whether the relationship between two categories varies as a function of another variable, the interpretation of β must be done in terms of an odd ratio. This index is obtained by making a ratio between the odds. Odd is expressed by $\pi_{i}/\left(1-\pi_{1}\right)$. Indeed, the standard outputs of the regression analysis model are represented by odd ratio or exp(β). In the case of the binomial logistic regression, the maximum likelihood (ML) algorithm is used to estimate the parameters. The log-likelihood function indicates how probable it is to obtain the expected value of the Y values of independent variables. For mathematical reasons, ML is multiplied by −2 and is expressed as −2LL. In the model containing both the intercept and independent variables, the value of the −2LL statistic represents the part of data variability that is not explained by the model: large and positive values indicate a low predictive capacity of the model. Another measure of adaptation of the model similar to the expected one is Chi-squared or $\chi^{2}$ (6).

(6) $$\chi^{2}=\sum_{i=1}^{k}{\frac{{\left(O_{i\;}-\;A_{i}\right)}^{2}}{A_{i}}}_{\;}$$

If $\chi^{2}$ coincides with 0, the observed frequencies correspond to the expectations. To check if there is a correlation between the observed and theoretical frequencies, and therefore to be able to exclude the null hypothesis (which means there is no correlation but is due to chance), $\chi^{2}$ must be higher than the tabular value present in the $\chi^{2}$ distribution tables for a *p*-value and degrees of freedom established. The degrees of freedom are expressed as (k−1). Other indices for “goodness fit” are: $R^{2}$ of Cox and Snell, which relates the likelihood of the model with the only intercept to the likelihood of the current model; $R^{2}$ of Nagerkelke, which is standardized to have a maximum of 1 (by comparing $R^{2}$ of Cox and Snell obtained on the current model to $R^{2}$ of Cox and Snell maximum). Higher values of $R^{2}$ are the evidence that observed frequencies almost correspond to those predicted.

In this study, the software used for data processing is SPSS v.25 (SPSS Inc., Chicago, IL, USA). The stepwise forward logistic regression analysis, consisting of a selection of independent variables per step with insertion test based on the significance of the score statistic and with removal test based on the probability of the Wald statistic, considering farm size, labor intensity, farmer’s age, intensity of information, level of education, and perceived complexity of the adoption process as independent variables.

## 5. Results and Discussions

### 5.1. Pilot Study

The data collected through the pilot study were content-analyzed to identify the behavioral, normative, and control beliefs underlying farmers’ awareness in adopting precision farming tools. Two research assistants repeatedly read the respondents’ answers and proposed a list of concepts potentially useful in creating a coding scheme. The assistants discussed each concept and modified their lists to converge on a common scheme that they used to codify all collected data. This analysis ultimately identified a set of beliefs that defined the variables of the model. Specifically, respondents noted that farm size, labor intensity, age, level of education, and information could affect, one way or the other, the adoption of precision farming tools. As for the events and/or situations that could facilitate vs. hinder adoption (complexity), respondents most frequently mentioned operational factors on the one hand; and on the other hand, the lack of knowledge and information that could help farmers to ensure a proper awareness in the adoption of precision farming.

### 5.2. Main Study

#### 5.2.1. Descriptive Results

A preliminary analysis showed that 28.7% of the respondents adopt PF technologies.

The reasons this value is higher than those estimated in other European countries, i.e. United Kingdom, Denmark’s rate is 10–15% [82], can be found in the type of sample interviewed. The respondents were all aware and interested in seeking information about precision farming technologies. 

The descriptive analysis showed different characteristics between adopters and non-adopters (Table 1). 

The adopters were characterized by an average farm size of 143 ha, whereas non-adopters 33.39 ha. The results show that PFTs’ adopter farmers are more likely to manage big farms. PFTs fit the model of a capital-intensive technology. In fact, they are characterized by high entry costs, overly “long payback period” (ROI); large fixed transaction ones, and other ‘hidden costs’, such as educational and informational ones [11,83,84,85]. Furthermore, if a farm has a technology system installed, the “switching cost” to a new technology might be onerous especially for small farms [11,14,59,86]. Larger farms that have a strong capacity to absorb costs and risks and are able to invest large amounts of capital, time, and learning in technologies, are more inclined to use PFTs [12,59,84,87,88,89,90]. In addition, their higher degree of division of labor and professional management may foster the willingness to invest in new technologies [56]. Most studies find a positive relationship between size and adoption [17,84,87,91,92,93,94,95]. Small farms could become PF adopters thanks to contractors or cooperation [96].

**Figure 1:**
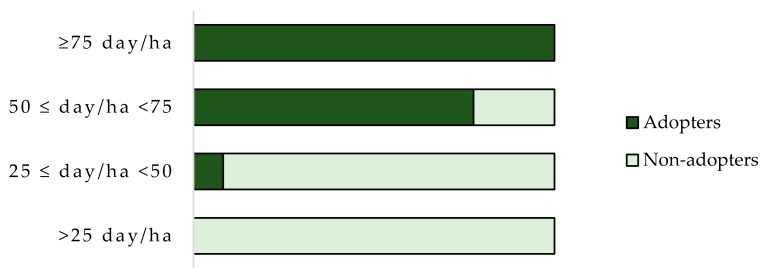
Percentage of precision farming tool adopters and non-adopters per labor intensity.

Figure 1 shows that labor intensity has a positive impact on adoption behavior. As pointed out by De Rose [97], the indicator of intensity of labor allows for the distinction between the areas where manual labor continues to be an important component of the production process in agriculture, and those where labor has been more widely replaced/flanked by automation. PF includes many automation and robotics technologies [98]. A high value of labor intensity is accompanied by a high level of PF adoption, where the role of technologies allows for the reduction of manual labor on the farm. 

The farms conducted by precision farming adopters report a higher intensity of labor (mainly >50 days/ha) compared to that of the non-adopters (mainly <50 days/ha), upholding the important role of technology in reducing labor hours. 

Age was found to have a negative impact on PFT adoption. Adopters were characterized by an average age of 43, whereas non-adopters had an average of 48 years. Several authors have verified that increasing age reduces the likelihood of PFT adoption. Young farmers, with longer planning horizons, may be more involved in more innovative farming [17,88,91,94,99,100].

**Figure 2:**
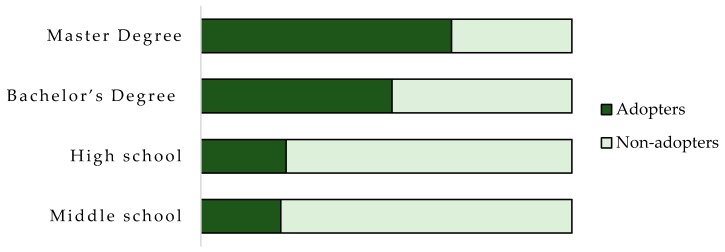
Percentage of PFT adopters and non-adopters per education level.

The positive relation between the level of education and PFT adoption is also demonstrated in Figure 2. Adopters are characterized by a high level of education; specifically, 62% of them had a master’s degree. In the scientific literature, it has been found that a high level of education (which can be measured in the number of years of formal education or ordinary education levels) is positively correlated with adoption [14,93,94,99,100,101,102]. PFT required a high level of human capital in term of capabilities and skills to manage and adapt better innovations for the specific farm levels [12,17,83,86,101,103]. This result is related to the previous one: young individuals are better educated and more technologically savvy in using high-technological practices for management decisions. They were found to have a greater capacity to decode new information and search for suitable tools to support production [99,100,101,104]. Older farmers that are less educated and more experienced, feel less necessity to invest or acquire information on emerging technologies [56,105]. 

**Figure 3:**
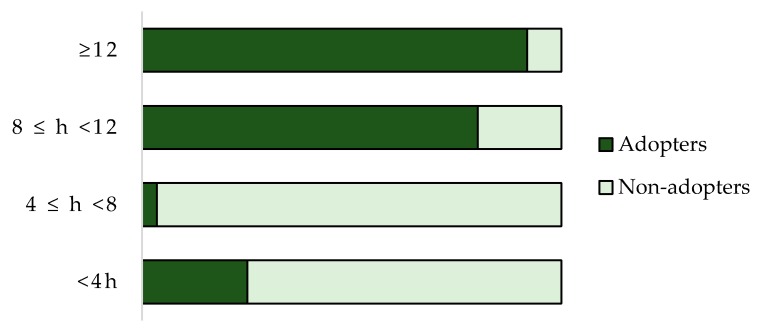
Percentage of PFT adopters and non-adopters per number of hours spent on information or formation activities.

Figure 3 shows that adopters are well-informed, and they spend more than eight hours/month on information and formation activities; non-adopters dedicate less than that. “Precision Farming is an information intensive activity” [106]. A farmer might opt for quick adoption if he is more likely to receive new information providing PFT. It is not easy to quantify the information intensity degree of farmers. It can be measured by considering access to information from a source (mass media or interpersonal communication such as consultants or extension services), or how often an individual receives the information within a period [83]. 

#### 5.2.2. Logit Regression Results

Table 2 shows a significative (<0.01 Pearson Correlation Index) relationship between independent variables, confirmed by the Pearson’s Chi-square test of socio-economic variables

The dependent variable is binomial and assumes value “0” if the farmer is a non-adopter, and value “1” if the farmer is an adopter of precision farming technologies. The classification table with intercepts only (Step 0) shows that 50% of the observations are correctly classified (Table 3).

On testing the model by including independent variables, the percentage of fairness increases. In fact, the model, whose prediction depends on the variables, correctly classifies 95% of the observations (Table 4).

Table 5 shows the estimates of the parameter B of the logistic model, standard error (S.E.), Wald level of significance, and exp(β) (defined as odd ratio). Based on the Wald test, the logic forward model selected up to two significant variables (<0.01) in two steps: perceived complexity and labor intensity. The probability of an individual to be an adopter is higher the lower the value of the perceived complexity variable is. As labor intensity increases, the probability of being an adopter increases. Indices of “goodness fit” of the model confirm that observed frequencies almost correspond to the predicted ones. Chi-squared has a statistical significance <0.001. The value of *R^2^* emphasizes on good fit to the data and, and therefore a good overall model fit.

The results show that a lower perceived complexity by farmers regarding different economic and organizational aspects, such as initial investment, long pay-back periods or role of expectations of farmers, and compatibility with traditional machinery, practices and management is associated with a greater adoption of Precision Farming Tools. 

The multi-step adoption process is influenced by many factors: socio-economic, agro-ecological, organizational, and institutional. Complexity is associated on one hand with organizational compatibility of new technologies to existing systems, and on the other with high initial investments and farmer expectations to get back the invested capital in a short time. The conducted analysis, filling a gap in the literature regarding PFT, shows that a greater perception of complexity can lead an aware individual not to acquire further information on new technologies and not to adopt it. The results also emphasize that labor intensity positively affects the adoption process. In high labor-intense farm, PFTs can serve as a complementary workforce that reduces the hours of manual work, leading the farmer to play other different roles and carry out greater tasks. Other factors, such as age, education, and intensity of information were not significant predictors in the analysis. The reason for this is the strong correlation between independent variables. Although the strong correlation, adequately tested, prevents the logit model from identifying all variables as significant, the descriptive results showed important different characteristics between adopters and non-adopters. As already demonstrated in recent scientific literature, young farmers with a high level of education, well-oriented to gathering information on PFTs, and with large farm sizes, are more likely to adopt or to continue to embrace PFTs [107,108,109,110].

## 6. Conclusions

The results of this study highlight the endogenous role of awareness in the adoption process. The latter primarily depends on factors influencing awareness, which is a prerequisite for adoption. Awareness mainly depends on the availability of information sources and on the quality of information provided to farmers [14,22,23]. A pertinent information policy will be key in ensuring a higher rate of PFT adoption among farmers. Agricultural policies will be decisive in promoting new measures that support information systems and networks or projects involving both small and large farms. New information-oriented political measures will lead to the increase in skills in agriculture and greater availability of technical professionalism and consultants. Greater levels of information among farmers can also reduce the perception of complexity involved in the adoption process. As the results of the study demonstrate, this perceived complexity has a significant role to play in convincing farmers to use PFTs, and is strongly linked to the level of information and education provided. Improving this level of information can help the farmer to understand the advantages and opportunities associated with precision agricultural instruments. Awareness of PFT benefits (e.g., reducing manual labor) can propel the farmer to adopt the technology. Policies over the years have pushed for adoption of innovation with economic incentives. This study confirms that while economic support is useful and important, especially in the Italian context characterized by small-medium farms (usually family-owned), other factors need to be taken into consideration. Future research should focus on innovations and solutions that offer environmental sustainability [111]. The combined actions of stakeholders in strengthening the role of information in the adoption process and proposing measures to foster dissemination of that information through innovation platforms [75] could help farmers gain more awareness of PFTs and their benefits, reduce the perception of complexity, and embrace the adoption. 

## Figures and Tables

**Table 1:** Characteristics of the interviewed farmers and their farms.

Variable	Adopters	Non-Adopters
**Farmer’s age**	43 years	48 years
**Level of education**		
Middle school	2%	7.3%
High school	12%	40.3%
Bachelor’s degree	24%	22.6%
Master’s degree	62%	29.8%
**Farm size**	143.36 ha	33.39 ha
**Labor intensity**		
>25 day/ha	0%	43.5%
25 ≤ day/ha < 50	4%	44.4%
50 ≤ day/ha < 75	42%	12.1%
≥75 day/ha	54%	0%
**Intensity of information**		
<4 h	10%	29.8%
4 ≤ h < 8	2%	54%
8 ≤ h < 12	52%	12.9%
≥12	36%	3.2%

**Table 2:** Correlation analysis results.

Variables	Perceived Complexity	Labor Intensity	Age	Education	Intensity of Information
Perceived complexity	1	−0.672 **	0.276 **	−0.449 **	−0.704 **
Labor intensity		1	−0.299 **	0.423 **	0.628 **
Age			1	−0.228 **	−0.329 **
Education				1	0.604 **
Intensity of information					1

Correlation indices are statistically significant at the 1% level (**).

**Table 3:** Classification table (Step 0).

Category	Predicted		Percentage
Observed	Non adopters	Adopters	
Non adopters	0	50	0
Adopters	0	50	100
Overall Percentage			50

**Table 4:** Classification table (Step 1).

Category	Predicted		Percentage
Observed	Non adopters	Adopters	
Non adopters	47	3	94
Adopters	2	48	96
Overall Percentage			95

**Table 5:** Output of logit model.

Variable	B	S.E.	Wald	Sig.	Exp(β)
Perceived Complexity	−16.359	6.464	6.404	0.011	0
Labor intensity	4.386	1.263	12.067	0.001	80.291
Constant	−0.201	3.639	0.003	0.956	
**Summary statistics**					
Likelihood ratio	24.586				
$R^{2}$ Cox and Snell	0.68				
$R^{2}$ Nagerkelke	0.907				
Chi-squared	114.043			0.000	

S.E. is the standard error of the parameter B; Sig. indicates the level of significance.
